# Application of Multidimensional Statistical Analysis in Tribotechnical Diagnostics of Hydraulic Fluids in Woodworking Equipment

**DOI:** 10.3390/ma14164628

**Published:** 2021-08-17

**Authors:** Michaela Hnilicová, Ján Turis, Richard Hnilica

**Affiliations:** 1 Department of Mechanics, Mechanical Engineering and Design, Faculty of Technology, Technical University in Zvolen, T.G. Masaryka 24, 960 01 Zvolen, Slovakia; michaela.hnilicova@gmail.com (M.H.); turis@tuzvo.sk (J.T.); 2 Department of Manufacturing Technology and Quality Management, Faculty of Technology, Technical University in Zvolen, T.G. Masaryka 24, 960 01 Zvolen, Slovakia

**Keywords:** tribology, analysis, predictive model, hydraulic fluid, woodworking

## Abstract

The article dealt with the assessment of the quality of hydraulic oil and determination of the mode of wear of the friction surfaces of Baljer & Zembrod manipulating lines through the information traces in the oils by applying tribotechnical diagnostics. We presented the assessment of the level of degradation of the oils. In addition, we presented the mode of wear of the friction surfaces washed in oil through evaluation of the qualitative and quantitative characteristics of the particles found in the oil. In detail, we focused on the application of suitable multivariate statistical methods on the data matrix. The article also presents predictive models that can sort oils into groups based on the assessment of quality of the oil and the state of the friction couples. The models can be used in research and in solving practical tasks in tribotechnical diagnostics of hydraulic fluids in woodworking equipment. Our results showed that the manipulation lines were greatly thermically stressed due to inadequate oil and machine maintenance. By correlative integration of all methods used, we could determine the real mode of the wear of the tribologic nodes of the machine. The experiment enabled the early detection of an undesirable process in the tribological node and implementation of corrective measures before the machine would break down.

## 1. Introduction

In the case [1], the physician analyzes the information obtained from the patient’s blood sample to detect any abnormal medical condition and make a diagnosis at the same time. A similar process takes place with machines. In this case, the tribotechnicians obtain the information measured in the lubricating oil for diagnosing and forecasting the machine.

To determine the technical condition of the machine, multiparametric diagnostics, which uses the results of several methods, is important and practically effective. If some of the used methods only state the existence of the malfunction, others can monitor its development, but tribodiagnostics is precisely the diagnostic method that monitors the development and, based on the detected state, provides the necessary information not only to prevent the malfunction but also to save money on future lubricant purchases [2].

According to [3], it is possible to obtain an overview of the technical condition of the mechanical system, wear of functional parts of the machine, deterioration and aging of oil, and localization of excessive wear, which is the cause of failures and system crashes. In this way, it is possible to sensitively observe the wear rate of the system as a function of time or in real time. It is also possible to check, for example, the condition of the filter systems, the tightness of the cooling systems, and so on. The authors [4] also note that a suitable interpretation of the results of the oil analysis will allow, in addition to early warning of the symptoms of the emerging failure, also to locate the place of occurrence of this failure.

Tribotechnical diagnostics deals with two major areas of problems: machine and oil. This statement is confirmed by [3], which shows that the analysis of lubricating oil is divided into two basic activities. Tribotechnical diagnostics of oil consists of determining the condition, prolonging the service life, and predicting the degradation of the lubricating oil in the machine, as well as assessing the magnitude of changes in the intensity and degree of degradation of oil to change its performance. Tribotechnical diagnostics of the machine is based on determining the mode, location, and trend of wear of the mechanical system (internal combustion engine, gearbox, hydraulic system, etc.) by evaluating the presence of foreign substances in the oil. Mechanical and ferromagnetic particles are analyzed from both a quantitative and qualitative point of view, by determining their composition, shape, quantity, and morphology.

By combining several classical and modern tribotechnical methods, it is possible to evaluate the technical condition of the machine and find out the reason for its wear, which passes a signal about its condition to the oil [5,6,7,8]. The authors of [9,10,11] state that the presence of thermal degradation products in oil causes unscheduled machine shutdowns, resulting in large losses. Using the methods of tribotechnical diagnostics, it was determined that the cause of excessive bearing wear is material fatigue due to cyclic motion [12,13]. Thermal degradation products present in the oil also have a major influence on the development of bearing fatigue wear, the presence of which also reduces the cooling efficiency, thus causing local overheating of the oil [10].

Although the oil can easily be pumped out and replaced with a new one, according to [14], the problem does not end there, and the resins will most likely reappear in a short time. The disposal of resins is more complicated than it would seem at first sight [15,16]. If an increased potential of the oil to form thermal degradation products is detected, it is necessary to quickly apply an oil cleaning method that is able to remove the degradation products from the oil in addition to the hard impurities. As the average size of these impurities is 0.08 µm, they cannot be captured by conventional filters. Electrostatic oil cleaning is a proven means of solving the complex cleaning of the entire hydraulic system. The author [17] states that electrostatic cleaning can remove any kind of contamination of any size from the oil. While conventional filtration methods achieve the capture of particles with a maximum size of 3 µm, the electrostatic method achieves oil cleanliness down to 0.05 µm.

In order to extend the life of the machine, to achieve maximum reliability of operation and to prevent the occurrence of a breakdown that would take on an emergency character, the authors [17,18] recommended to use an electrostatic cleaning system in combination with bypass filtration technology to remove thermal degradation products in the lubrication system of the manipulating lines.

The authors [19,20,21] point out the importance of performing a complex analysis of oil and machine condition by introducing multiparametric diagnostics, which includes tribotechnical diagnostics and its implementation in the machine maintenance plan. A proactive approach ensures a constant check of the machine’s technical condition and increases its operability [22,23]. The cost of implementing a proactive maintenance program is compensated by savings in maintenance costs.

The aim of the presented manuscript is to reveal the interrelationships and structure in the monitored tribodiagnostic parameters of individual samples of hydraulic oil using appropriate mathematical and statistical methods.

## 2. Materials and Methods

**Figure 1:**
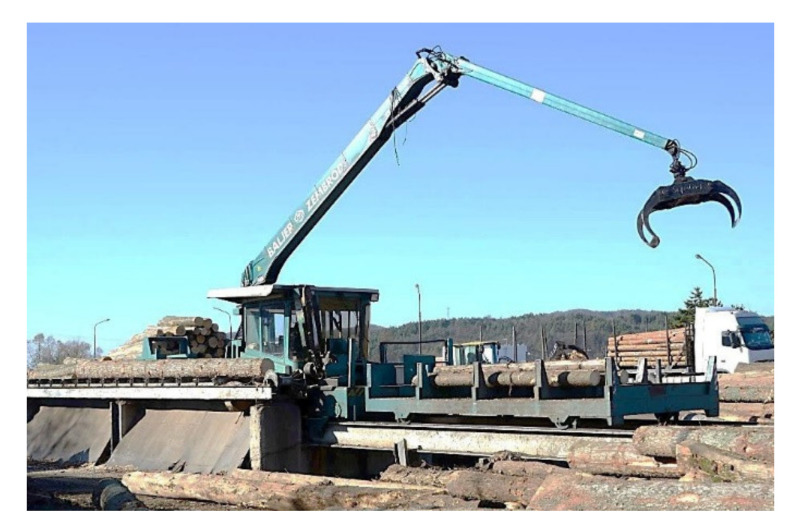
Baljer & Zembrod manipulating line.

For the machine to work well, it must be properly designed, well made, and properly lubricated, taking into account the choice of a suitable lubricant. In order for the lubricant to meet the predetermined requirements, it must be checked and treated regularly. Many manufacturing companies hire workers who deal with the measurement and evaluation of vibration and with the care of oils. As part of cost-saving measures, this care is linked only to refilling the lubrication system, but not so much to evaluating the lubricant, which is the essence of the problem. This is the main reason why we chose the Baljer & Zembrod log manipulating sorting trolley (Figure 1) working in the Kriváň Branch Plant (hereinafter referred to as the manipulating line) as the object of research. This is where hydraulic oil care only involves filling up the oil, changing fluid, filters, and cleaning the oil tank. They are less interested in the information that lubricant research can provide them using tribotechnical diagnostic methods.

The manipulating line is a rail vehicle with a mounted crane and a saw, which performs the division, shortening, and sorting of logs. All elements of the working and moving performance are performed electrohydraulically. The required power is provided by one electric motor. The hydrostatic mechanism, consisting of a grouping of hydraulic and other elements through which the pressure fluid circulates, consists of two open and two closed hydraulic circuits. The manipulating line is filled with hydraulic oil marked AGIP OSO 46, which is manufactured by a company named Eni (Eni S.p.A., Roma, IT, Italy). According to the technical norm [24], the liquid is classified as HLP 46 and ISO-L-HM 46 according to the technical norm [25]. Due to its high viscosity index, this mineral oil guarantees faultless operation, even with strong temperature fluctuations. The low freezing point ensures that the device is immediately ready for use, even in low temperatures. In addition, these oils have excellent wear protection and very good oxidative stability, as well as excellent anti-corrosion properties. The basic parameters of the oil are given in Table 1.

In order to compare the results, we carried out the research on two manipulating lines filled with the same filling, of which line 081 was put into operation in 2006 and line number 072 was put into operation in 2008.

In order to obtain correct information on the state of oil quality and the condition of the machine, representative samples were collected by following the basic procedures of dynamic sampling according to [27,28,29,30].

For sampling, we used sample cards made of HDPE (High-density polyethylene) material with a volume of 250 mL, which we clearly marked with basic data about the oil and the machine. We rinsed the sample cards several times with a suitable solvent (Petrolether) before collection and left them closed until the moment of collection.

The recommended time required for the circulation and perfect dispersion of all particles present in the oil was determined from relation (1) according to [1], by adding the value of the volume of oil in the system and its flow, which is given in the master sheet of the manipulating line as follows:

(1) $$t_{c}=3\cdot \left(V/Q\right)$$

where t_c_ is the oil circulation time in the system (mins), V is the system volume (L), and Q is the flow (L min^−1^).

Although the recommended particle circulation time in the oil is 6 min, the machine has been in operation for at least two hours before sampling. Samples were taken while the machine was running at an operating temperature of approximately 55 °C. All samples were performed from the primary sampling site, i.e., from an oil tank, by a suction sampling method using a vacuum sampler with a Bürkle model MiniSampler PE suction tube (Bürkle GmbH, Bad Bellingen, D, Germany). The sample boxes with the collected oil were stored in a dark and cool place.

The hydraulic oil and the machine were monitored for one year, between two hydraulic changes, with a sampling frequency of approximately 500 operating hours. Table 2 and Table 3 provide information on the dates of hydraulic oil extraction, the operating time of the manipulating lines, and information on adding oil to the system.

In order to evaluate the oil quality and the state of wear of the friction surfaces of the manipulating lines, through the information in the oil, the focus had to be on evaluating the degree of oil degradation (aging) and wear regime of the machine friction surfaces by analyzing impurities and metal elements in the oil. The parameters that were measured and determined were divided into three groups:

- Degradation (aging) of hydraulic oil:
  − Kinematic viscosity at 40 °C → capillary viscometer and Julabo ME-16G Visco Bath (JULABO GmbH, Seelbach, D, Germany);
  − Acid number → Schott TitroLine alpha 05 plus (Schott Instruments GmbH, Mainz, D, Germany);
  − Analysis of oxidation products, aromatic products, and low-temperature antioxidant by infrared spectroscopy → Thermo Nicolet Avatar 330 spectrometer (Thermo Nicolet Corporation, Madison, WI, USA);
  − Potential of the oil to form thermal degradation products → vacuum filter unit with electric pump Vacuubrand MZ 1C (VACUUBRAND GMBH + CO KG, Wertheim, D, Germany).
- Contamination of hydraulic oil:
  − Water content → Schott TitroLine KF trace (Schott Instruments GmbH, Mainz, D, Germany);
  − Number and classification of the shape of mechanical impurities → laser particle shape classifier and particle counter Spectro Scientific LaserNet Fines-C (SPECTRO CS spol. s r.o., Ostrava, CZ, Czech Republic);
  − Analysis of contaminating elements by atomic emission spectroscopy → device for emission spectrometry with inductively coupled plasma.
- Wear of the friction surfaces of the machine:
  − Analysis of particles from wear using atomic emission spectroscopy and ferrographic analysis → ferrography laboratory Spectro T^2^FM Q^500^ (AMETEK Spectro Scientific, Chelmsford, MA, USA).

Lubricant analyses were performed in cooperation with the specialized tribotechnical laboratory InterTriboDia, s.r.o. in Tlmače and with the Technical Faculty of the Czech University of Life Sciences in Prague.

We evaluated the measured values using mathematical or statistical methods. Our goal was to reveal the interrelationships and structures in the monitored parameters and sampling of individual samples. To uncover these links, we used an analysis of the main components. We also decided to compare the two manipulating lines with each other by non-parametric Mann–Whitney U-test, looking for differences in the parameters observed. Finally, we compiled mathematical models by applying discriminatory analysis, which was able to distinguish between high-quality oil, oil with increased degradation, and oil with a high degree of degradation but also between the operational and limit wear mode of the friction pairs of the manipulating line.

By applying the exploratory analysis of multidimensional data, they became acquainted with the source data matrix and its structure, while we chose the following procedure in the analysis:

- We compiled a source data matrix X (n × m), containing n = 10 objects and m = 13 characters.
- Using a box plot, we assessed the variability of the data set and revealed the existence of outlying and extreme values.
- We compiled a correlation matrix and a matrix graph of character correlation, in which we observed the interdependencies between individual characters and the similarity between objects.

As part of the data preparation, we also focused on testing the normality of the data, which we verified with the following basic tests of one-dimensional normality: Kolmogorov–Smirnov and Lilliefors test, Shapiro–Wilks test, normal probability plot, and histogram.

When detecting the bonds in the monitored parameters and collecting individual samples by the method of the main components, we used the following procedures:

- We have reduced the number of characters in the matrix from thirteen to m = 9.
- We numbered the main components and determined the number of main components based on the following rules:
  − The selection of the main components according to the Kaiser criterion included only those components whose eigenvalues λi ≥ 1 in the model, and we neglected those whose eigenvalues were less than 1,
  − The main components had to explain 70% to 90% of the variability of the source matrix,
  − Based on the Cattel index graph of the base of eigenvalues, we determined the break point between the vertical wall and the horizontal bottom.
- We calculated the custom vectors for the main components and determined the first and second main components as follows:
  − The first main component captured most of the variability of the data, as it described the most information,
  − The second main component then covered most of the variability that was not included in the first main component.
- With component loads and a graph of component loads, we expressed the relationship between the original features of the source matrix and the main components.
- By the scatter plot of the component score, we expressed the coordinates of the objects in the space of the main components and looked for connections between the individual objects.

We tested the significance of the differences of the compared manipulating lines using the non-parametric Mann–Whitney test. We used the following procedure for testing:

- We chose the levels of significance α, for which we looked for tabular critical values of the Mann–Whitney test and formulated the basic and alternative hypothesis.
- We arranged the data by size from the smallest to the largest value and assigned a serial number to each value.
- We added up the serial numbers of both machines so that the condition applies:

(2) $$P_{1}+P_{2}=n\left(n+1\right)/2$$

where P_1_ is the sum of the orders of the first machine, P_2_ is the sum of the orders of the second machine, and *n* is the sum of all the orders.

We calculated the test statistics for both machines individually according to the following relationships:

(3) $$U_{1}=n_{1}\cdot n_{2}+n_{1}\left(n_{1}+1\right)/2-P_{1}$$

(4) $$U_{2}=n_{1}\cdot n_{2}+n_{2}\left(n_{2}+1\right)/2-P_{2}$$

where *U*_1_ is the test statistic of the first machine, *U*_2_ is the test statistic of the second machine, *n*_1_ is the sum of the orders of the first machine, and *n*_2_ is the sum of the orders of the second machine.

- The smaller value of the calculated tested statistics was chosen as the test criterion *U* = min (*U*_1_, *U*_2_). Then, we decided on the test result, i.e., on rejecting, or not rejecting the null hypothesis by comparing the value of the test criterion with the critical value of the Mann–Whitney test.

In the last part, we focused on data processing using discriminant analysis, which classified the objects into the appropriate group. We used the following testing procedure to compile the predictive models:

- We proposed the classification of the oil quality status into classes and assigned them the appropriate designation as follows:
  − Quality oil: the oil is able to fulfill its function in the machine (oil condition expressing its quality (KO = 0)),
  − Oil limit state: oil with a worsened level of oxidative and thermal degradation (KO = 1),
  − Critical oil level: oil with a high level of oxidative and thermal degradation (KO = 2).
- We excluded features with high abnormality and low variability from the analysis.
- We chose the dependent variable and the independent variables. We marked the KO character as a dependent variable, which determined the association to the given group.
- By applying discriminant analysis, we also gradually selected the features that contributed the most to discrimination. For the model, we selected only those discriminants that acquired the highest value of the Wilks’ lambda criterion and the largest value of the F-test criterion. Discriminators with a tolerance of less than 0.001 were not included in the model.
- We determined standardized coefficients of the canonical function, by which we proposed discriminant functions.
- By calculating the coordinates of individual characters, we divided objects into classes.
- We displayed a scatter plot to assess the share of the proposed functions in group resolution.
- We determined centroids, which are border coordinates of individual groups.
- We displayed a classification matrix in which we monitored the number of objects included in individual groups and the predictive ability of the proposed model, which depends on the number of objects included in the correct classes.

We proceeded in the same way when applying discriminant analysis in the tribotechnical diagnostics of machines. We marked the character SO (the degree of wear of the friction pairs of the machine) as a dependent variable, which determined the appropriation to the given group as follows:

- Normal wear mode: indicates a condition where particles are released by wear in small quantities, and small dimensions (SO = 0) begin to form in the monitored machine.
- Excessive wear regime: indicates that particles are formed in larger quantities, larger dimensions, and different morphology (SO = 1).

## 3. Results

We started the evaluation of the condition of hydraulic oils using tribotechnical diagnostics by inspecting the machines on site. We were identifying the degradation and contamination of oils based on the color, transparency, and odor of individual samples taken.

**Figure 2:**
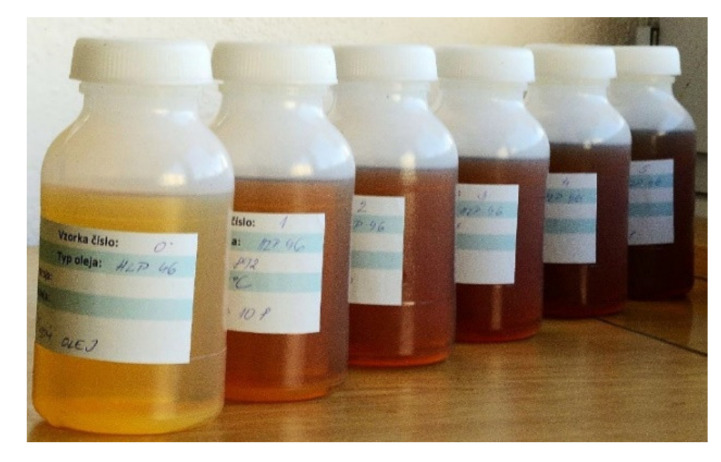
Visual evaluation of the color and appearance of hydraulic oil samples.

During a visual inspection of the machines on site, we noticed that there were oil leaks in the manipulating lines through seals. This finding was matched by the frequent addition of oil to the system, which is shown in Table 2 and Table 3. By observing the color and appearance of the collected oils (Figure 2), we concluded that the oils change color rapidly (from light straw to dark brown), which could be the result of rapid degradation (aging) of the oil. Otherwise, the oils appeared to be transparent, without turbidity and without much contamination. A change in the oils also occurred when their odor was identified. Hydraulic oils gradually lost their natural odor, and we noticed an acidic odor, which we assigned to oxidized or, in the worst case, to overheated oil.

**Figure 3:**
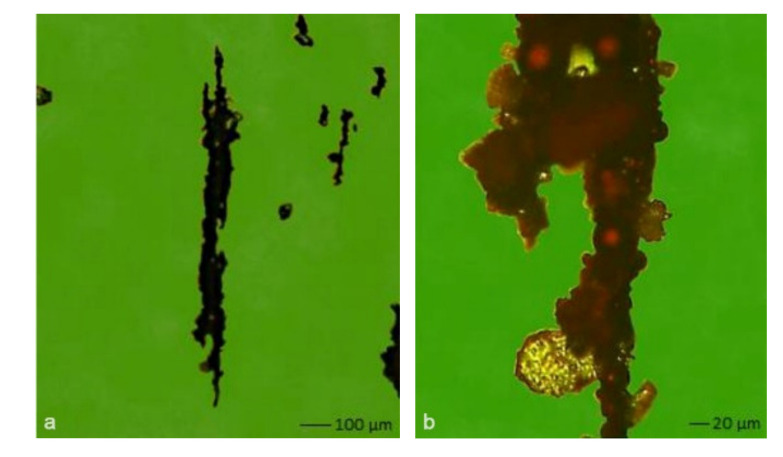
Identified dark metal oxides in oil: (**a**) clusters of dark oxides; (**b**) robust spherical particles of size 60 µm.

Various particles were identified in hydraulic oil samples—abrasive, adhesive, fatigue, non-metal, fiber, and unclassified. The results of the distribution of mechanical impurities obtained by means of an automatic laser particle computer did not signal an unfavorable wear regime of oil-washing friction pairs during operation of the manipulating line 072. Non-magnetic oxidized particles in a chain were identified by ferrographic analysis (Figure 3). A significant increase of small magnetic fatigue particles (up to 15 µm) was recorded in the fourth sampling. The presence of spherical particles (up to 5 µm) identified the on-start of bearing fatigue.

**Figure 4:**
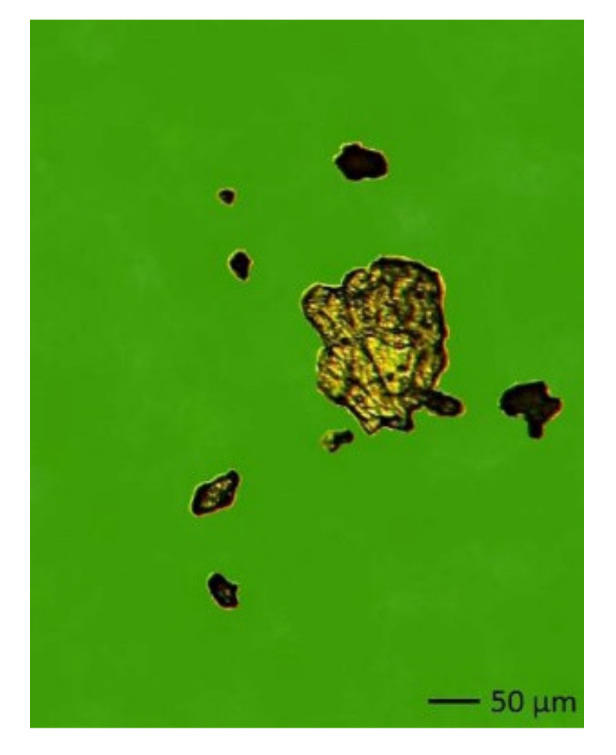
Magnetic and non-magnetic fatigue particles.

Lamar particles (up to 40 µm) and a large amount of black metal oxides were identified in the oil samples of manipulating line 081. The presence of non-magnetic oxidized particles confirmed that excessive heat generation occurred in the machine. The origin of the damage to the friction node of the machine was confirmed by the presence of magnetic and paramagnetic fatigue particles of 60 to 130 µm (Figure 4) on the ferromagnetic trace in the fifth sampling.

In the first step, we became acquainted with the source matrix of data X (n × m), which is given in Table 4. The matrix contains n = 10 samples of hydraulic oil HLP 46 from manipulating lines Baljer & Zembrod, in which we monitored m = 13 selected tribodiagnostic parameters. For the purposes of applying the statistical methods of the selected method, the individual oil samples were called objects and tribodiagnostic parameters features.

The matrix is supplemented by a code designation of the oil level (KO) next, which expresses the quality of hydraulic oil in terms of ability to perform the prescribed functions for which it was intended and a code designation of the machine condition (SO), which expresses the degree of wear of machine friction pairs washed with this oil as follows: 0—quality oil, or normal state of wear, 1—oil limit state, or excessive wear regime, and 2—critical oil condition.

As some characters had a large variance compared to others, we had to modify the data before applying the statistical method. This form of adjustment is called data standardization, which also removed the dependence on physical units. Characters and objects had zero value and variance *s*^2^ = 1 after standardization. We standardized the data using a box plot, which revealed the existence of remote and extreme values. Due to the same construction of manipulating lines 072 and 081, which also have the same oil filling, we applied statistical analysis for both machines together.

**Figure 5:**
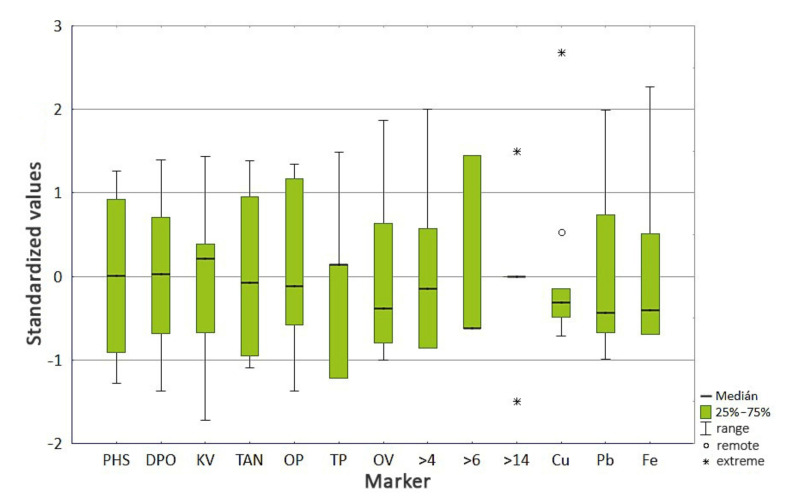
Box plot of standardized data.

Figure 5 shows the variability of each character, remote (○) and extreme (∗) objects. The box plot shows that the sign >14 and Cu had a high abnormality. The extreme value of the purity class, which expresses the number of particles larger than 14 µm, exceeded the permitted limit values in the object S_1_-5 and S_2_-5. These values should be excluded from the selection. The remote value of Cu did not exceed the limit value in its content, which is why we have kept it in the selection for the time being. We decided to exclude these features from the principal component analysis and from the discriminant analysis by testing normality.

Table 5 presents the correlation matrix of all features, which we used to observe the structures between the characteristics of oil degradation, oil contamination, and wear of the friction surfaces of the machine and the degree of dependence between them.

By correlation analysis, we have found a statistical insignificance between the features characterizing the degradation of oil and the features characterizing the condition of the machine. On the contrary, at the level of significance α < 0.05 (orange and at the same time red color), the features characterizing the degradation of oil significantly correlated with each other; for example, TAN significantly correlated with KV, OP, and TP. In the correlation matrix, we also observed the interdependence between the features characterizing the state of the machine (oil pollution and wear of the machine friction pairs), where, for example, the Cu feature significantly correlated with the OV, >4, Pb, and Fe features.

**Figure 6:**
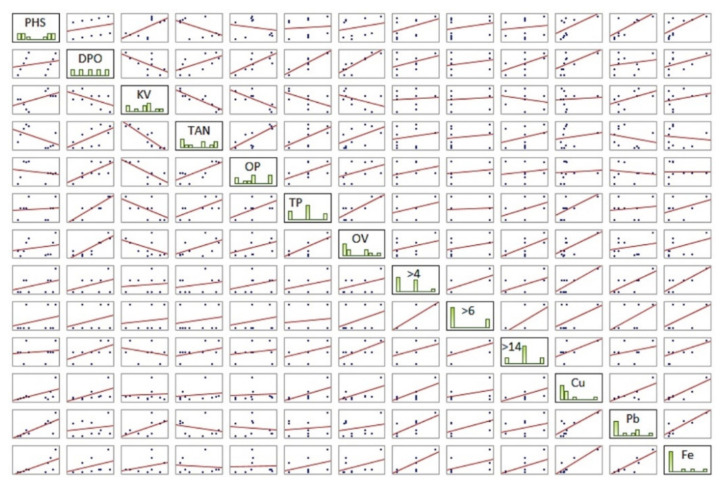
Character correlation matrix graph.

A clearer form of the correlation matrix is the character correlation matrix graph, which is compiled from scatter plots, for all pair combinations of characters (Figure 6). It was possible to read the deviating points influencing the pairwise correlation coefficient and the similarities of the characters between the objects, from the matrix graph, by visual assessment of the differences of the created scattering diagrams. Higher values of the correlation coefficient were clearly recognizable in the scatter plot by grouping objects into a straight line (for example, correlation of Fe with Cu). Conversely, lower values of the correlation coefficient led to object scattering (e.g., PHS correlation with >14).

Many statistical methods assume that the data being used come from a normal distribution. Since the discriminant analysis we will work with later divides the objects into groups of the file, we tested the normality for each file separately. Therefore, we divided the individual characters into independent and dependent variables. We determined KO and SO as dependent variables, by which we determined the affiliation to the given file. The classification of individual oil samples into the appropriate set is coded in Table 4.

We decided whether the data had a normal distribution or not by applying the Kolmogorov–Smirnov, Lilliefors, and Shapiro–Wilks test. We first performed a data file normalization, which was focused on tribotechnical diagnostics of oil. We chose the characters PHS, DPO, KV, TAN, OP, and TP as independent variables. We decided on the KO character as a dependent variable. The test results for all groups together (KO = 0, 1, 2) are shown in Table 6. The *p*-value of the Lilliefors test was decisive for us in the evaluation. The results in the table did not reject the hypothesis that each character came from a normal distribution at the significance level α = 0.05.

When testing the normality of the dataset, which was focused on the tribotechnical diagnostics of the machine, we chose the characters PHS, DPO, >4, >6, >14, Cu, Pb, and Fe as independent variables. In Table 7, with the dependent variable SO, we see that the borderline values of the Lilliefors *p*-value of the variables >4, >6, >14, Cu, and Fe were lower than the significance level α = 0.05. This, together with the result of the S-W test, rejected the hypothesis of the normality of these features at the significance level of 5%. Normality is very difficult to prove for variables expressing a purity class, as they do not directly express the actual number of particles but the code of the relevant size class. Therefore, we considered the >4 and >6 characters to be normally distributed. Based on the result of the box plot in Figure 5, we have already said that the characters >14 and Cu have a strong abnormality. As these features did not even show one-dimensional normality and could cause problems in estimating the discriminant function, we excluded them from the discriminant analysis as well as from the analysis of the main components.

We decided on the normality of the Fe feature by visual assessment of the normality with a normal probability graph (N-P plot) and a graphical estimate of the probability density—a histogram.

**Figure 7:**
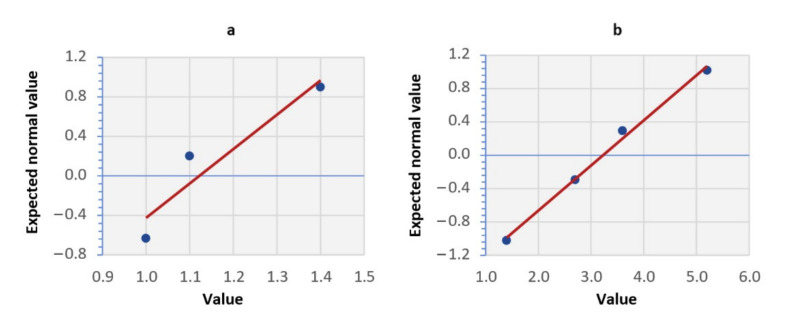
Normal probability graph of the Fe attribute: (**a**) results for the group SO = 1; (**b**) results for the group SO = 0.

By representing the N-P fence (Figure 7), we evaluated that the curve determined by the points of the graph is a straight line, so we could consider the Fe sign to be normally distributed.

**Figure 8:**
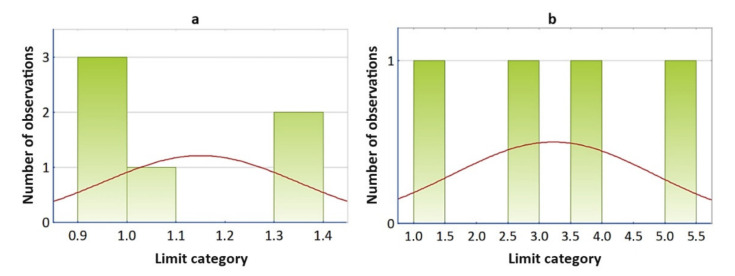
Attribute histogram Fe: (**a**) results for the group SO = 1; (**b**) results for the group SO = 0.

As stated by the appearance of the graphs showing the density of probability (Figure 8), according to which we assessed the similarity with the density curve of the theoretical distribution with the Gaussian curve, we could not unequivocally claim that the Fe sign comes from the normal distribution.

When comparing the results of all tests for the Fe characteristic, we took into account that a small deviation from normality does not have a significant effect on the results of the discriminant analysis and the analysis of the main components. In this case, we therefore considered this information to be normal, and we continued to work with it without modification.

Using the principal components analysis method, we analyzed the relationships and structures between characters and objects. Our goal was to find the features that most affect the operating condition of the hydraulic oil and to reveal the similarity of objects to each other. In order for the analysis of the main components to be performed regularly (positively defined correlation matrix), we could observe less than ten characters for ten objects. Therefore, we excluded the features PHS, DPO, >14 and Cu from the analysis, thus reducing the variables included in the model of the main components from thirteen to *m* = 9.

Table 8 shows the eigenvalues of the correlation matrix. The variance percentage and the cumulative percentage describe the variability in the original characters and at the same time describe the relevant main components. According to Kaiser’s criterion, we selected only those main components that have their own number *λ_i_* ≥ 1.

Next, we described the relative importance of each feature with respect to the main component by calculating the eigenvectors (component weights). These vectors are coefficients of the equation that allow you to combine pre-normalized characters.

Based on the results, we described the importance of the characters with respect to the main component as follows:

- The most important feature of the first main component was the TP feature;
- The most important feature of the second main component was the Pb feature.

**Figure 9:**
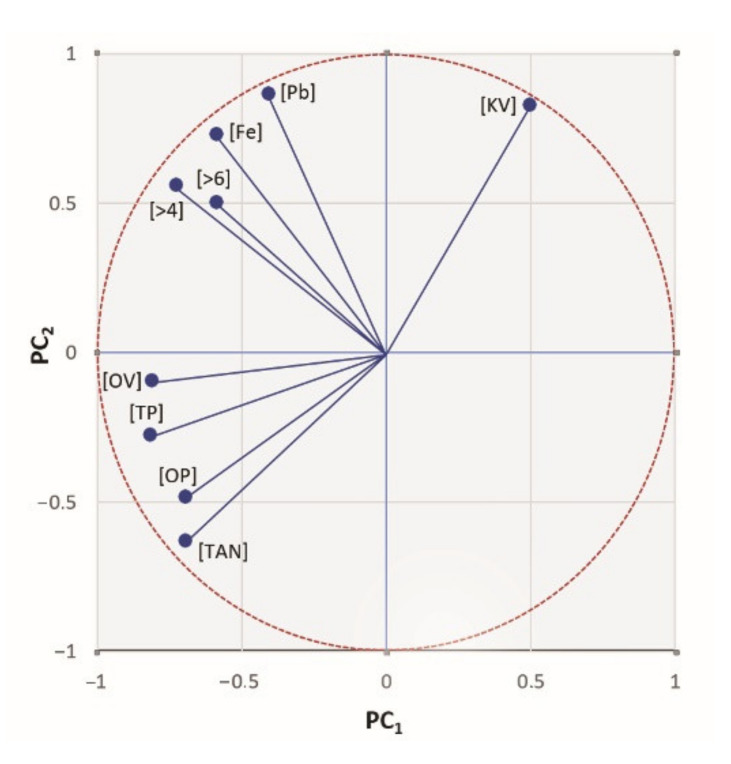
Loading plot.

The graph of component loads showed us to what extent the individual original features contributed to the first main component or to the second main component (Figure 9).

Characters that are placed in the graph in the same direction against the beginning of the graph interact and are positively correlated. Then, characters placed in the opposite direction are negatively correlated with them. From Figure 9, we could say that all features except the kinematic viscosity were positively correlated with each other, but at the same time, they were not correlated with the kinematic viscosity. This is due to the fact that the kinematic viscosity is not a very objective parameter determining the quality of the oil, as its value was influenced by the amount of oil that was added to the system.

From the results of the eigenvectors of the correlation matrix and the graph of the component score, we could clearly say that the first major component was the degradation of hydraulic oil, which is focused on oil quality. The main parameters that negatively affected the quality of the oil were thermal products but also the acid number and oxidation products. High levels of thermal products have affected the wear of the machine’s friction surfaces, leading to the formation of wear particles, mechanical impurities, and imperfect lubrication of the integrating machine particles. The second main component thus distinguished between oil contamination and the wear of the friction pairs of the machine, of which the wear had a greater weight. This statement was also supported by a strong positive correlation between >4, >6, and Fe.

**Figure 10:**
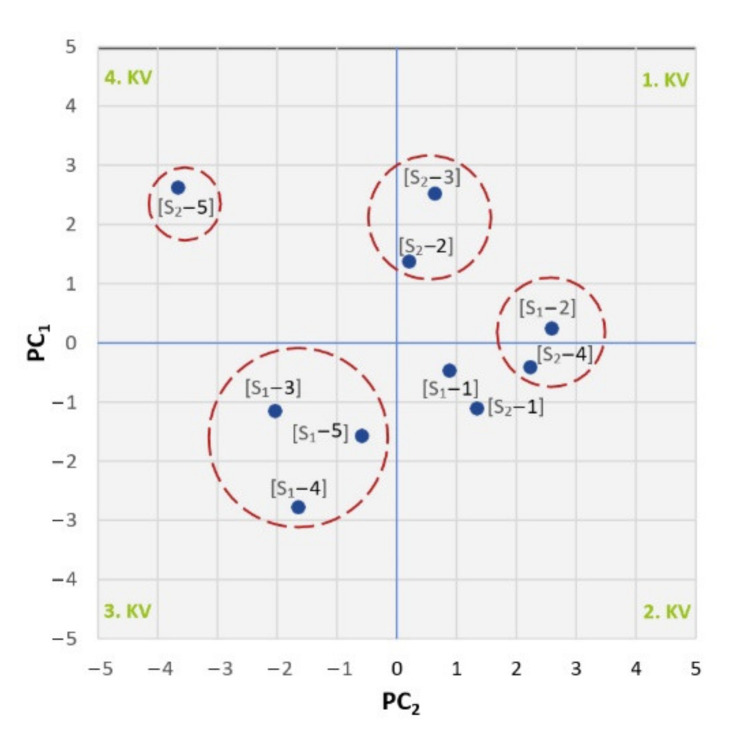
Scatter plot of component scores.

In the last step, we used the scatter plot of the component score, which is graphically shown in Figure 10, to look for deeper connections between the individual objects.

By diagnosing the scatter plot, we could observe that objects that are similar to each other formed clusters in the scatter plot. Examples were objects S_1_-1 and S_2_-1, which were dissimilar to most of the other objects due to the fact that all the measured values satisfied the operating conditions. Close to these objects were objects S_1_-2 and S_2_-4, which had all other measured parameters within the standard except for elevated levels of thermal degradation. Therefore, these objects could be considered to be broadly similar. Objects S_1_-3, S_1_-4, and S_1_-5 were outlying objects predominantly thermo-oxidatively stressed. Objects S_2_-2 and S_2_-3 were similar in increasing proportions of copper, lead, and iron in the oil, and thus increasing amounts of mechanical impurities. Feature S_2_-5 was a solitary and remote feature that was strongly dissimilar to the other features. This object represented an oil that had ceased to perform its prescribed functions due to a critical level of degradation products and, from the point of view of machine diagnostics, an excessive wear regime was occurring in the oil.

Next, we observed the equality of the observed parameters of the compared manipulating lines. Since some traits did not have a normal distribution of random variables, we decided to use the non-parametric Mann–Whitney U-test to evaluate the obtained data. The test did not directly observe the measured values, but it did discern their ordinal location after sorting them into the input series.

Before applying the statistical method, we selected significance levels *α*. In the tables of critical values for the Mann–Whitney test, we looked up the critical values for these levels for the number of first and second machine extractions n_1_ = n_2_ = 5:

- If the significance level α = 0.01 → critical value U_α_ = 0;
- If significance level α = 0.05 → critical value U_α_ = 2.

Based on the result, we selected a significance level of α = 0.05 for diagnosis and formulated a base and alternative hypothesis:

- If the test criterion U = min(U_1_, U_2_) ≤ 2 → we reject the null hypothesis H_0_ of equality of values of the observed parameters between the first and the second machine;
- If the test criterion U = min(U_1_, U_2_) >2 → we cannot reject the null hypothesis H_0_ of equality of values of the observed parameters between the first and the second machine.

Next, we present a procedure for monitoring the equality of acid number values between the manipulating lines. We arranged the measured acid number values of both machines in ascending order in a single variation series and assigned a serial number to each value, regardless of which machine it came from. Equally large values were assigned a so-called average order. Table 9 shows the ordinal numbers of the mixed selection acid number values.

We computed the sums of the rank orders of the first machine P_1_ = 38 and the second machine P_2_ = 17, as well as the test statistics according to relations (3) and (4). We used the smaller of the values of the calculated test statistics as a test criterion and compared it with the tabulated critical value of the Mann–Whitney test at the significance level of α = 0.05. The calculated test criterion (U = min(2) ≤ 2) was equal to the critical value at the α = 0.05 significance level; therefore, we rejected the null hypothesis of equality of the observed acid number parameter for both machines.

Based on the resulting values (Table 10) of the test criteria calculated using the Mann–Whitney U-test, we found that manipulating lines 072 and 081 differ from each other in the values of kinematic viscosity and also in the values of acid number and lead concentration.

In terms of tribotechnical oil diagnostics, which focuses on oil quality, our aim was to find a mathematical model that would allow us to classify oil samples into groups and thus assess whether or not an oil with given measured values is able to perform its function in the machine. The oils were classified into groups according to how the range of measured values of the monitored parameters varied with respect to the established warning and critical limit values.

Discriminant analysis assumes that the data being worked with come from a normal distribution. Data that do not show a normal distribution could negatively affect the discriminant functions. Furthermore, multicollinearity in the data, meaning that two or more discriminants are highly correlated, could affect the result. By assessing the box plot of the standardized data (Figure 5), the feature selection correlation matrix (Table 5), and verifying the normality of the data, we excluded the PHS and KV features from the dataset due to low linearity.

We selected only those discriminants in the model that contributed the most to the grouping and eliminated those that were not appropriate for the analysis. Table 11 lists the DPO and TP discriminants that were identified by the discriminant function analysis as the most significant and thus met the inclusion criterion.

We found eigenvalues whose corresponding eigenvectors represented the coefficients of the canonical function. We had three groups of code designations, so we got two eigenvalues and two canonical functions. The result of the Chi-square test of successive roots (Table 12) indicated the statistical significance of the discriminant function (*p*-value = 0.003 for the first root and 0.06 for the second root).

We determined the standardized coefficients of the canonical function, which we fit to the equation of the canonical function. The canonical discriminant functions had the form:

(5) $${\mathrm{DF}}_{1,\mathrm{KO}}=-1.51405\cdot X_{\mathrm{DPO}}+0.70726\cdot X_{\mathrm{TP}}$$

(6) $${\mathrm{DF}}_{2,\mathrm{KO}}=1.02336\cdot X_{\mathrm{DPO}}-1.68505\cdot X_{\mathrm{TP}}.$$

**Figure 11:**
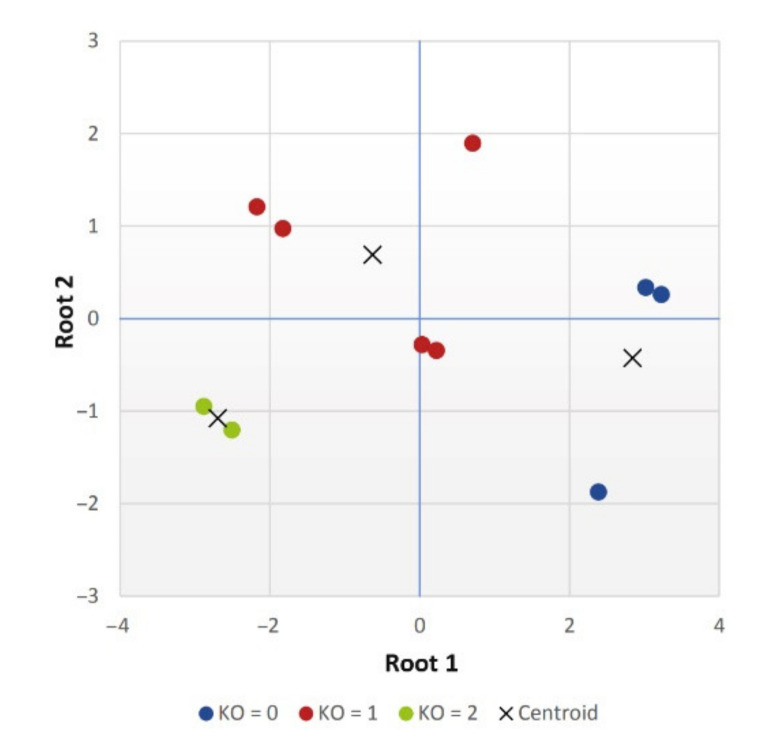
Scatter diagram.

By fitting the standardized values of the individual traits to both functions, we determined the coordinates of each group in two-dimensional space, the so-called canonical scores. By displaying a dot plot (Figure 11), we judged whether both functions contributed to the resolution of the groups or whether only one was sufficient.

From Figure 11, we could tell that the first discriminant function distinguished, although not significantly, the first group from the other two. Both of the proposed discriminant functions were needed to distinguish between the second and third groups.

We also plotted the coordinates of the centroids of each group in two-dimensional space in the dot plot, which determined the boundary coordinates of each group. An object was classified into the group for which it achieved the smallest Mahalanobis distance from the centroids of the groups. The centroids of each group are listed in Table 13.

The analysis also resulted in a classification matrix (Table 14) in which we tracked the number of objects assigned to each group.

By the classification matrix, we confirmed that all objects were classified into the appropriate class, thus achieving 100% classification and full predictive ability of the proposed models.

By applying discriminant analysis, we also classified the objects into the appropriate group by fitting a suitable model and thus decided whether or not excessive wear of the oil-washed friction pairs of the machine occurred in the object under study.

By assessing the box plot of the standardized data (Figure 5), the feature selection correlation matrix (Table 5), we excluded features >14 and Cu from the dataset due to low variability. Verifying normality (Figure 7 and Figure 8), we retained the Fe feature in the dataset. By discriminant function analysis, the result of which is shown in Table 15, the features OV, >6, and Fe were selected into the model.

For the two groups of code designations, we obtained one eigenvalue and one canonical function. Calculation of the Chi-squared test in Table 16 indicated statistical significance of the canonical discriminant function *p*-value = 0.007.

The canonical discriminant function, which we constructed from the standardized coefficients of the canonical function, had the form:

(7) $${\mathrm{DF}}_{\mathrm{SO}}=0.72391\cdot X_{\mathrm{OV}}-1.01734\cdot X_{>6}-0.95559\cdot X_{\mathrm{Fe}}.$$

By fitting the standardized values of the individual traits to the canonical function, we determined the numerical value of the function called the canonical score, which we compared with the centroids—the cutoff points of the individual groups. The centroids of each group are shown in Table 17.

Since we had a straight-line view, we determined a border centroid by which we divided the set into two groups:

(8) $$C=\left(C_{1}+C_{2}\right)/2=1.69332-2.53998/2=-0.42333.$$

If the inequality DF_SO_ > C was valid, i.e., if the canonical score was greater than the value of the threshold centroid, we classified the object in the first group into the second group.

**Figure 12:**
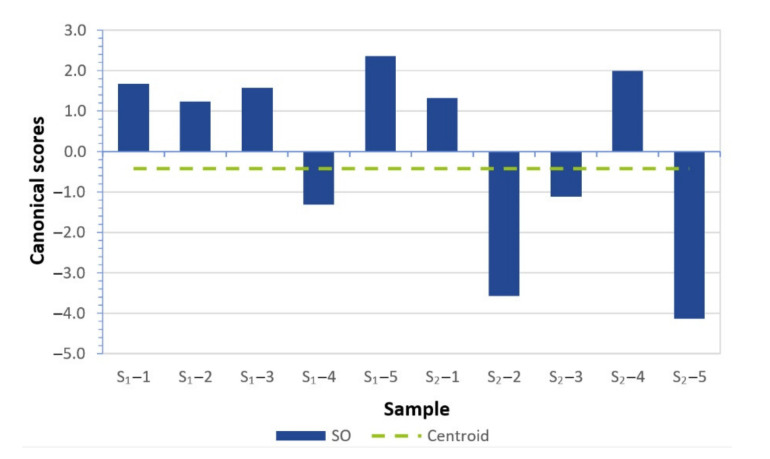
Discriminant function values for individual machines.

A point plot representation was not possible in this case, because we obtained a straight line representation via a single canonical function. Figure 12 shows the resulting values of the discriminant function for each machine.

In the classification matrix shown in Table 18, we tracked the number of objects classified into each group.

The classification matrix achieved 100% predictive ability of the proposed model.

## 4. Discussion

By correlation analysis (Table 5), we found statistical insignificance between the measured parameters characterizing oil degradation and the features characterizing the machine condition. On the contrary, from the diagnostic point of view, the significance of the parameters between each other was found.

By analyzing the main components, we were able to find the features that most affect the operational condition of hydraulic oil. The dominant parameters are thermal products. The component captures the maximum variability of the source matrix (the largest part of the variance) and therefore describes the most information. The first main component is oil degradation focusing on oil quality, which is mainly influenced by thermal products. We do not consider kinematic viscosity as an objective tribodiagnostic parameter to determine oil quality, since its values are influenced by the amount of oil added to the system. In addition to the acid number and oxidation products, the critical degree of thermal degradation also affects the distribution of mechanical impurities and the concentration of chemical elements present in the oil. Thus, the second principal component distinguishes between oil contamination and the wear of the machine’s friction pairs, of which wear has the greater variability.

By scatter plot of the component scores (Figure 10), we expressed the coordinates of the oil samples in the principal component space and revealed the similarity of the objects to each other. In the first quadrant, the samples whose increasing amounts of wear particles indicate an excessive wear regime of the friction pairs of the oil-washed machines are located. In the second quadrant, the samples are operable, with low levels of oxidative and thermal degradation; on the contrary, the third quadrant contains samples highly thermally and mainly oxidatively stressed. Finally, the fourth quadrant contains an object that has failed to perform the prescribed functions due to a critical level of degradation products and, from the point of view of machine diagnostics in oil, an excessive wear regime. From a tribotechnical point of view, the operation of this facility cannot be considered trouble-free.

By a non-parametric Mann–Whitney U-test, we found that the difference in the values of the observed parameters between manipulating lines 072 and 081 is mainly in the kinetic viscosity parameters but also in the acidity number and lead concentration. By the mentioned statistical method, we also observe that the manipulating lines are most equal in water content and in the level of thermal products present in the oil. Last but not least, it can also be observed in the results that manipulating line 072 is more oxidatively stressed compared to the other manipulating line, which, however, experiences an excessive wear regime. The difference in the behavior of the manipulating lines may be influenced both by the operator and by the different age of the machines.

We were unable to distinguish samples that are serviceable, marginal, or critical in terms of oil quality and machine wear mode by the scatter plot. Therefore, in the last step, our goal was to build mathematical models that could classify the oil samples into predetermined groups. By discriminant analysis, we built a model aimed at tribotechnical oil diagnostics that can distinguish between high-quality oil, low degraded oil, and highly degraded oil. The second model, which was focused on tribotechnical machine diagnostics, distinguishes between samples with normal and limiting wear regimes. By correctly classifying the oils into groups, which was performed at 100%, we confirmed the capability of the proposed models. Therefore, we consider the models to be functional, but they do not account for cases where the level of thermal products does not increase with increasing hours worked. Such a condition can only be achieved by long-term monitoring of the oil quality and the degree of wear of the machine by tribotechnical diagnostic methods, as well as by ensuring proper maintenance.

The inclusion of a new oil sample in the appropriate group, which expresses the quality of the oil, is possible by creating two new variables in the program STATISTICA (version 12). With the first variable, we calculate the canonical score of this case, and with the second variable created, we get the classification of the case into the appropriate group according to the calculated canonical score. Inserting the new case into the computer and recalculating the new variables created is enough. To assign the new oil sample to the appropriate group, which expresses the wear mode, is determined by the Mahalanobis distance from the centroids of the groups. The sample is assigned to the group for which this distance is the smallest. In both cases, the success rate of the new classification is to be determined by showing the efficiency of the canonical discriminant function.

## 5. Conclusions

Discriminant analysis is one of the more complex and demanding statistical methods used in many sectors, mostly in medicine, economics, chemistry, or sociology [31,32,33,34,35,36,37,38,39,40,41]. So far, multidimensional statistical methods have not found a more significant application in the field of tribotechnics. However, by applying discriminant analysis in the realized experiment, we confirmed its use in the processing of measured data in tribotechnical diagnostics. The analysis of the main components, which can be used as spatial analysis for discriminant analysis, is also important.

Based on the interpretation of the results of tribotechnical methods and in cooperation with users of manipulating lines, we found that the machines overheated locally during operation due to the presence of thermal degradation products contained in the oil. Their presence caused oil leaks and reduced the efficiency of the machine’s cooling system.

By regularly evaluating the condition of the hydraulic oil by the oil analysis program, it is possible to obtain a larger number of measured samples. Such long-term monitoring will allow more operational conditions to be taken into account in the future. This will lead to a more comprehensive statistical evaluation of the experiment. Further implementation of the research will make it possible to monitor the quality of the oil and the condition of the friction pairs of the manipulating line, which is filled with ecological vegetable-based oil.

Experience shows that knowledge of machines in which the surfaces of solid bodies interact in their relative motion should be combined with several scientific disciplines into multiparametric diagnostics. Therefore, we recommend that we further apply the results of tribotechnical diagnostics or confront them with the results of vibrodiagnostic and thermodiagnostic measurements of hydrogenerators and hydromotors located in manipulating lines.

Due to the critical number of resins contained in the oil, we advised the machine operator not to extend the time limit set by the manufacturer for oil change, which is set at 2000 operating hours worked. We also recommended regularly carrying out comprehensive maintenance of the cooling system, regularly carrying out analysis and evaluation of the condition of the hydraulic oil and the machine, as well as to treat oil and lubrication systems with electrostatic filtration equipment in combination with bypass filtration technology. The results of the application of these recommendations may be subject to further research.

## Figures and Tables

**Table 1:** Basic physical and technical characteristics of the hydraulic oil OSO 46 [26].

Characteristics	Value
Viscosity at 40 °C [mm^2^ s^−1^]	45
Viscosity at 100 °C [mm^2^ s^−1^]	6.8
Viscosity Index	100
Pour point [°C]	−27
Acid number [mg KOH g^−1^]	0.59

**Table 2:** Sampling intervals hydraulic oil HLP 46 manipulating line 072.

Sampling	Date of Sampling	Machine Operating Hours [h]	Oil Operating Time [h]	Adding Oil
Oil change	25 April 2013	15,400	0	4 June 2013 +10 L
1	24 June 2013	15,892	492	9 July 2013 +10 L
2	28 August 2013	16,392	992	20 September 2013 +15 L
3	31 October 2013	16,907	1507	-
4	14 January 2014	17,389	1989	1 March 2013 +20 L
5	18 March 2014	17,882	2482	-
Oil change	3 April 2014	18,011	2611	-

**Table 3:** Sampling intervals hydraulic oil HLP 46 manipulating line 081.

Sampling	Date of Sampling	Machine Operating Hours [h]	Oil Operating Time [h]	Adding Oil
Oil change	25 April 2013	20,400	0	-
1	24 June 2013	20,916	516	14 August 2013 +10 L
2	28 August 2013	21,421	1021	21 October 2013 +30 L
3	31 October 2013	21,887	1487	18 December 2013 +30 L
4	14 January 2014	22,313	1913	-
5	18 March 2014	22,800	2400	-
Oil change	3 April 2014	22,924	2524	-

**Table 4:** Source data matrix.

Marker	S_1_-1	S_1_-2	S_1_-3	S_1_-4	S_1_-5	S_2_-1	S_2_-2	S_2_-3	S_2_-4	S_2_-5
PHS	15,892	16,392	16,907	17,389	17,882	20,916	21,421	21,887	22,313	22,800
DPO	492	992	1507	1989	2482	516	1021	1487	1913	2400
Oil degradation										
KV	46.5	46.5	46.3	46.1	46.0	46.8	46.9	46.6	46.6	46.6
TAN	0.72	0.78	0.82	0.85	0.84	0.68	0.68	0.69	0.70	0.79
OP	100	123	179	174	177	100	137	138	135	136
TP	2	3	3	3	4	2	2	3	3	4
Oil contamination										
OV	63	57	63	73	78	58	59	59	73	85
>4	17	17	18	18	17	17	18	18	17	19
>6	16	16	16	17	16	16	17	16	16	17
>14	13	13	12	14	13	12	13	13	13	14
Wear of the abrasive surfaces of the machine										
Cu	1.0	1.2	1.3	1.5	1.4	1.3	1.1	2.1	1.4	4.0
Pb	1.0	1.0	1.2	1.5	1.2	2.0	2.2	2.1	1.2	2.9
Fe	1.0	1.0	1.1	1.4	1.4	1.0	2.7	3.6	1.4	5.2
KO	0	1	1	1	2	0	0	1	1	2
SO	0	0	0	1	0	0	1	1	0	1

PHS: operating hours of the machine [h]; DPO: oil operating time [h]; KV: viscosity at 40 °C [mm^2^ s^−1^]; TAN: acid number [mg KOH g^−1^]; OP: oxidation products [%]; TP: thermic products; OV: water content [ppm]; >4: purity class expressing a number of particles larger than 4 µm; >6: purity class expressing a number of particles larger than 6 µm; >14: purity class expressing a number of particles larger than 14 µm; Cu: copper concentration [ppm]; Pb: plumbum concentration [ppm]; Fe: iron concentration [ppm]. ─ warning limit values, resp. critical values.

**Table 5:** Character selection correlation matrix.

Marker	PHS	DPO	KV	TAN	OP	TP	OV	>4	>6	>14	Cu	Pb	Fe
PHS	1	0.247	0.604	−0.540	−0.183	0.102	0.227	0.405	0.294	0.145	0.551	0.771	0.687
DPO	0.247	1	−0.568	0.616	0.731	0.908	0.859	0.425	0.310	0.548	0.54	0.191	0.422
KV	0.604	−0.568	1	−0.882	−0.729	−0.587	−0.457	0.090	0.105	−0.234	0.081	0.504	0.307
TAN	−0.540	0.616	−0.882	1	−0.749	−0.672	0.538	0.209	0.185	0.341	0.161	−0.264	−0.106
OP	−0.183	0.731	−0.729	−0.749	1	0.601	0.424	0.332	0.216	0.178	0.053	−0.129	0.012
TP	0.102	0.908	−0.587	−0.672	0.601	1	0.768	0.345	0.093	0.452	0.618	0.125	0.411
OV	0.227	0.859	−0.457	0.538	0.424	0.768	1	0.378	0.391	0.632	0.638	0.244	0.416
>4	0.405	0.425	0.090	0.209	0.332	0.345	0.378	1	0.724	0.477	0.758	0.752	0.819
>6	0.294	0.310	0.105	0.185	0.216	0.093	0.391	0.724	1	0.690	0.445	0.617	0.544
>14	0.145	0.548	−0.234	0.341	0.178	0.452	0.632	0.477	0.690	1	0.546	0.314	0.528
Cu	0.551	0.554	0.081	0.161	0.053	0.618	0.638	0.758	0.445	0.546	1	0.755	0.884
Pb	0.771	0.191	0.504	−0.264	−0.129	0.125	0.244	0.752	0.617	0.314	0.755	1	0.869
Fe	0.687	0.422	0.307	−0.106	0.012	0.411	0.416	0.819	0.544	0.528	0.884	0.869	1

─ significance at α < 0.01; ─ significance at α < 0.05.

**Table 6:** Normality test (KO: all groups).

Marker	Test Statistics	Kolmogorov–Smirnov *p*-Value Test	Lilliefors Test *p*-Value	Shapiro–Wilk Test	*p*-Value
PHS	0.213960	*p* > 0.20	*p* > 0.20	0.867130	0.092540
DPO	0.138358	*p* > 0.20	*p* > 0.20	0.934938	0.498176
KV	0.214015	*p* > 0.20	*p* > 0.20	0.938662	0.538222
TAN	0.195689	*p* > 0.20	*p* > 0.20	0.872006	0.105489
OP	0.226085	*p* > 0.20	*p* < 0.15	0.885910	0.152441
TP	0.253902	*p* > 0.20	*p* < 0.10	0.832503	0.035864

*n* = 10. ─ warning limit values, resp. critical values.

**Table 7:** Normality test (SO: all groups).

Marker	Test Statistics	Kolmogorov–Smirnov *p*-Value Test	Lilliefors Test *p*-Value	Shapiro–Wilk Test	*p*-Value
PHS	0.213960	*p* > 0.20	*p* > 0.20	0.867130	0.092540
DPO	0.138358	*p* > 0.20	*p* > 0.20	0.934938	0.498176
OV	0.251466	*p* > 0.20	*p* < 0.10	0.873081	0.108564
>4	0.304586	*p* > 0.20	*p* < 0.01	0.780895	0.008489
>6	0.432720	*p* < 0.05	*p* < 0.01	0.594174	0.000047
>14	0.300000	*p* > 0.20	*p* < 0.01	0.814840	0.021948
Cu	0.358425	*p* < 0.15	*p* < 0.01	0.644782	0.000190
Pb	0.249908	*p* > 0.20	*p* < 0.10	0.873239	0.109024
Fe	0.358411	*p* < 0.15	*p* < 0.01	0.739474	0.002657

*n* = 10. ─ warning limit values, resp. critical values.

**Table 8:** Eigenvalues correlation matrix.

Component *i*	Eigenvalue *λ_i_*	% Dispersion	Cumulative %
1	3.931	43.67	43.67
2	3.260	36.22	79.90
3	0.816	9.06	88.96
4	0.486	5.40	94.36
5	0.216	2.40	96.76
6	0.110	1.23	97.99
7	0.099	1.10	99.09
8	0.077	0.85	99.95
9	0.005	0.05	100.00

**Table 9:** Serial numbers of the acid number parameter.

Value	0.68	0.68	0.69	0.70	0.72	0.78	0.79	0.82	0.84	0.85
Machine	2	2	2	2	1	1	2	1	1	1
Sequence	1.5.	1.5.	3.	4.	5.	6.	7.	8.	9.	10.

1 cross-cutting line 072; 2 cross-cutting line 081.

**Table 10:** The resulting values of test criteria.

Marker	Test Statistics *U*_1_	Test Statistics *U*_2_	Test Result
KV	25	0	Reject *H*_0_
TAN	2	23	Reject *H*_0_
OP	8.5	16.5	Do not reject *H*_0_
TP	10.5	14.5	Do not reject *H*_0_
OV	11.5	13.5	Do not reject *H*_0_
>4	16	9	Do not reject *H*_0_
>6	15	10	Do not reject *H*_0_
>4	22	12.5	Do not reject *H*_0_
Cu	17	8	Do not reject *H*_0_
Pb	23	2	Reject *H*_0_
Fe	20	5	Do not reject *H*_0_

─ warning limit values, resp. critical values.

**Table 11:** Discriminant functional analysis (oil quality).

Marker	Wilks’ Lambda	Partial Lambda	F-Test (2.5)	*p*-Value	Tolerance	1-Toler. (R^2^)
DPO	0.299320	0.279878	7.718967	0.021923	0.299437	0.700563
TP	0.164871	0.508111	2.904221	0.131183	0.299437	0.700563

*n* = 10. ─ warning limit values, resp. critical values.

**Table 12:** Chi-square test (oil quality).

Roots	Eigenvalue	Canonical Correlation	Wilks’ Lambda	Chi-Square	Degrees of Freedom	*p*-Value
0	5.825778	0.923849	0.083773	16.11770	4	0.002865
1	0.748816	0.654358	0.571815	3.63310	1	0.056641

**Table 13:** Centroids of individual groups (oil quality).

Group	Root 1	Root 2
Centroid 1: KO = 0	2.84415	−0.42816
Centroid 2: KO = 1	−0.62792	0.68811
Centroid 3: KO = 2	−2.69643	−1.07803

**Table 14:** Classificatory matrix (oil quality).

Group	% positive	KS = 0 *p* = 0.3	KS = 1 *p* = 0.5	KS = 2 *p* = 0.2
KO = 0	100	3	0	0
KO = 1	100	0	5	0
KO = 2	100	0	0	2

**Table 15:** Discriminant functional analysis (machine condition).

Marker	Wilks’ Lambda	Partial Lambda	F-Test (2.5)	*p*-Value	Tolerance	1-Toler. (R^2^)
OV	0.213588	0.734272	2.171362	0.191034	0.601395	0.398605
>6	0.414108	0.378721	9.842779	0.020136	0.711932	0.288068
Fe	0.340239	0.460946	7.016699	0.038084	0.700122	0.299878

*n* = 10. ─ warning limit values, resp. critical values.

**Table 16:** Chi-square test (machine condition).

Roots	Eigenvalue	Canonical Correlation	Wilks’ Lambda	Chi-Square	Degrees of Freedom	*p*-Value
0	5.376260	0.918242	0.156832	12.04178	3	0.007241

**Table 17:** Centroids of individual groups (machine condition).

Group	Root 1
Centroid 1: SO = 0	1.69332
Centroid 2: SO = 1	−2.53998

**Table 18:** Classificatory matrix (machine condition).

Group	% Positive	SO = 0 *p* = 0.6	SO = 1 *p* = 0.4
SO = 0	100	6	0
SO = 1	100	0	4

## Data Availability

All the data is available within the manuscript.

## References

[1] Peťková V., Stopka J., Pačaiová H., Balla J., Kureková M., Demian P., Sloboda A., Kmec P., Lošonský M. (2012). Tribotechnika v Teórii a Praxis.

[2] Peťková V. (2012). Tribodiagnostika Oleja a Tribotechnika Stroja?.

[3] Sejkorová M. (2013). Metody Tribotechnické Diagnostiky.

[4] Žarnovský J., Peťková V., Ružbarský J. (2009). Diagnostika Strojov a Zariadení.

[5] Kučera M., Čuchran J., Hnilicová M. Možnosti využitia moderných tribotechnických metód pre hodnotenie oterových kovov v použitých mazacích olejoch. Proceedings of the Mob. Energetické Prostriedky-Hydraul-Zivotn. Prostr-Erg. Mob. Prostried.

[6] Hnilicová M., Stanovský M., Kučera M., Hnilica R. Tribotechnická analýza hydraulických olejov lesných kolesových traktorov. Proceedings of the Použitie Ekol. Vhodných Médií V Hydraul. A Maz. Systémoch Lesn. Strojov.

[7] Hnilicová M., Kučera M. (2013). Tribotechnická diagnostika hydraulických olejov v laboratórnych a prevádzkových podmienkach. Acta Fac. Tech..

[8] Hnilicová M., Kučera M., Aleš Z. Systematizácia hodnotenia prevádzkových vlastností výrobných mechanizmov na manipulačných skladoch dreva. Proceedings of the XVI Mezinárodní Vědecká Konference Mladých.

[9] Nováček V. (2011). Monitorování potenciálu oleje k tvorbě úsad v olejových systémech. TriboTechnika.

[10] Phillips W. (2006). The high-temperature degradation of hydraulic oils and fluids. J. Synth. Lubr..

[11] Beran E., Łoś M., Kmiecik A. (2008). Influence of thermo-oxidative degradation on the biodegradability of lubricant base oils. J. Synth. Lubr..

[12] Peťková V., Kureková M. (2012). Čo je nepriateľom strojných zariadení. Slovgas.

[13] Romanowicz P.J., Szybiński B. (2019). Fatigue Life Assessment of Rolling Bearings Made from AISI 52100 Bearing Steel. Materials.

[14] Litwin W. (2016). Influence of local bush wear on water lubricated sliding bearing load carrying capacity. Tribol. Int..

[15] (2019). DuPont Ion Exchange Resins. Treating Oil Contaminated Ion Exchange Resins with an Industrial Surfactant.

[16] Ilyushin P.Y., Lekomtsev A.V., Ladeyshchikova T.S., Rakhimzyanov R.M. (2018). The efficiency assessment of the “cold flow” method against the deposition of asphaltenes, resins and paraffins. Perm J. Pet. Min. Eng..

[17] Štollmann V. (2010). Nezabúdajme na hydraulické systémy. TriboTechnika.

[18] Baboo P. (2016). Advancement in Oil Filtration with Electrostatic Oil Cleaner.

[19] Hnilicová M., Kučera M. Proaktívna údržba výrobných strojov založená na analýze mazacích olejov. Proceedings of the XIV Medzinárodná Vedecká Konferencia Mladých.

[20] Kučera M., Kopčanová S., Sejkorová M. (2020). Lubricant Analysis as the Most Useful Tool in the Proactive Maintenance Philosophies of Machinery and its Components. Manag. Syst. Prod. Eng..

[21] Nembhard A., Sinha J., Pinkerton J., Elbhbah K. (2014). Combined vibration and thermal analysis for the condition monitoring of rotating machinery. Struct. Health Monit..

[22] Rakyta M., Fusko M., Herčko J., Závodská Ľ., Zrnić N. (2016). Proactive approach to smart maintenance and logistics as a auxiliary and service processes in a company. J. Appl. Eng. Sci..

[23] Mačužić I., Jeremič B. (2004). Proactive approach to oil maintenance strategy. Tribol. Ind..

[24] DIN 51524-2:2006 (2017). Pressure Fluids—Hydraulic Oils—Part 2: HLP Hydraulic Oils; Minimum Requirements.

[25] STN EN ISO 6743-4:2002 (2002). Mazadlá, Priemyselné Oleje a Podobné Výrobky (Trieda L)—Klasifkácia—Časť 4: Skupina H (Hydraulické Systémy).

[26] ENI AGIP OSO: Catalog Sheet. https://webserver.flak.no/vbilder/02603_2.pdf.

[27] Dálik P. (2011). Laboratórne analýzy používaných olejov. TriboTechnika.

[28] Fitch B. (2014). Anatomy of Representative Oil Sample: Part 1—Sample Bottles. Mach. Lubr..

[29] Peťková V. (2012). Tribotechnická diagnostika ako účinný nástroj proaktívnej údržby. Slovgas.

[30] Oil Analyzers, Inc (2001). Oil Analysis Services.

[31] Zhichao G., Dongxue Z., Zhihui H., Lanrong C. (2018). A Preliminary Study on Discriminant Analysis of Syndrome Types in the Recovery Period of Stroke in Traditional Chinese Medicine. BioMed Res. Int..

[32] Bhuyan K.C. (2018). A Note on the Application of Advanced Statistical Methods in Medical Research. Biomed. J. Sci. Tech. Res..

[33] Popović B.V. (2014). Understanding advanced statistical methods. J. Appl. Stat..

[34] Sueyoshi T., Goto M. (2012). Efficiency-based rank assessment for electric power industry: A combined use of Data Envelopment Analysis (DEA) and DEA-Discriminant Analysis (DA). Energy Econ..

[35] Rodrigues L., Rodrigue L. (2018). Economic-financial performance of the Brazilian sugar cane energy industry: An empirical evaluation using financial ratio, cluster and discriminant analysis. Biomass Bioenergy.

[36] Ștefan R.-M. (2012). A Comparison of Data Classification Methods. Procedia Econ. Financ..

[37] Wanga C., Zhoua R., Huanga Y., Xiea L., Yinga Y. (2019). Terahertz spectroscopic imaging with discriminant analysis for detectingforeign materials among sausages. Food Control.

[38] Podsiadlo P., Stachowiak G.W. (2005). Development of advanced quantitative analysis methods for wear particle characterization and classification to aid tribological system diagnosis. Tribol. Int..

[39] Yanga H., Irudayaraj J., Paradkar M.M. (2005). Discriminant analysis of edible oils and fats byFTIR, FT-NIR and FT-Raman spectroscopy. Food Chem..

[40] Alvarez-Guerra M., Ballabio D., Amigo J.M., Bro R., Viguri J.R. (2010). Development of models for predicting toxicity from sediment chemistryby partial least squares-discriminant analysis and counter-propagationartificial neural networks. Environ. Pollut..

[41] Yakymova L., Kuz V. (2019). The use of discriminant analysis in the assessment of municipal company´s financial health. Econ. Sociol..

